# 4-*n*-Butyl-3-(3-methyl­phen­yl)-1*H*-1,2,4-triazol-5(4*H*)-one

**DOI:** 10.1107/S1600536808019661

**Published:** 2008-07-05

**Authors:** Tashfeen Akhtar, Shahid Hameed, Muhammad Zia-ur-Rehman, Tanveer Hussain Bukhari, Islamullah Khan

**Affiliations:** a Department of Chemistry, Quaid-i-Azam University, Islamabad 45320, Pakistan; b Applied Chemistry Research Centre, PCSIR Laboratories Complex, Lahore 54600, Pakistan; c Chemistry Department, Government College University, Lahore, Pakistan

## Abstract

In the mol­ecule of the title compound, C_13_H_17_N_3_O, the two rings make a dihedral angle of 56.63 (13)°. Mol­ecules are linked by strong N—H⋯O inter­molecular hydrogen bonds into chains running along the *c* axis.

## Related literature

For related literature, see: Akhtar *et al.* (2006 ▶, 2007 ▶, 2008 ▶); Aoyama *et al.* (1984 ▶); Al-Masoudi *et al.* (2006 ▶); Demirbas *et al.* (2002 ▶); Lin *et al.* (2005 ▶); Torres *et al.* (2005 ▶); Witkowski *et al.* (1972 ▶).
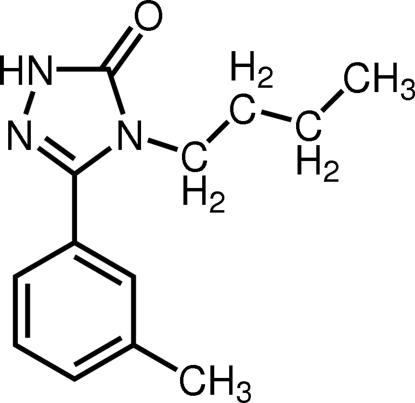


## Experimental

### 

#### Crystal data

C_13_H_17_N_3_O
*M* *_r_* = 231.30
Orthorhombic, 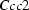

*a* = 16.905 (5) Å
*b* = 18.139 (5) Å
*c* = 8.145 (2) Å
*V* = 2497.5 (11) Å^3^
*Z* = 8
Mo *K*α radiation
μ = 0.08 mm^−1^
*T* = 296 (2) K
0.26 × 0.19 × 0.12 mm

#### Data collection

Bruker APEXII CCD area-detector diffractometer
Absorption correction: none
8129 measured reflections
1791 independent reflections
1118 reflections with *I* > 2σ(*I*)
*R*_int_ = 0.066

#### Refinement

*R*[*F* ^2^ > 2σ(*F* ^2^)] = 0.046
*wR*(*F* ^2^) = 0.106
*S* = 1.07
1791 reflections
160 parameters
1 restraint
H atoms treated by a mixture of independent and constrained refinement
Δρ_max_ = 0.15 e Å^−3^
Δρ_min_ = −0.25 e Å^−3^

### 

Data collection: *APEX2* (Bruker, 2007 ▶); cell refinement: *APEX2*; data reduction: *SAINT* (Bruker, 2007 ▶); program(s) used to solve structure: *SHELXS97* (Sheldrick, 2008 ▶); program(s) used to refine structure: *SHELXL97* (Sheldrick, 2008 ▶); molecular graphics: *ORTEP-3 for Windows* (Farrugia, 1997 ▶) and *PLATON* (Spek, 2003 ▶); software used to prepare material for publication: *WinGX* (Farrugia, 1999 ▶) and *PLATON*.

## Supplementary Material

Crystal structure: contains datablocks I, global. DOI: 10.1107/S1600536808019661/bt2734sup1.cif

Structure factors: contains datablocks I. DOI: 10.1107/S1600536808019661/bt2734Isup2.hkl

Additional supplementary materials:  crystallographic information; 3D view; checkCIF report

## Figures and Tables

**Table 1:** Hydrogen-bond geometry (Å, °)

*D*—H⋯*A*	*D*—H	H⋯*A*	*D*⋯*A*	*D*—H⋯*A*
N2—H22⋯O1^i^	0.94 (3)	1.84 (3)	2.775 (3)	170 (3)

Symmetry code: (i) 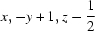.

**Fig. 1.**
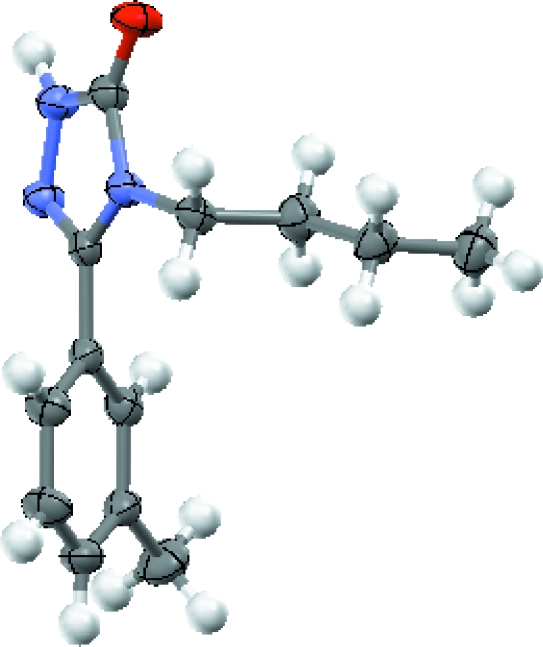
The asymmetric unit of the title compound showing the intramolecular hydrogen bond. Displacement ellipsoids are drawn at the 50% probability level.

**Fig. 2.**
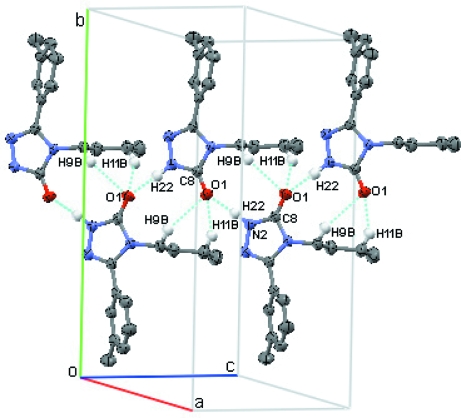
Perspective view of the three-dimensional crystal packing showing hydrogen-bonds and other intermolecular interactions (dashed lines). H atoms not involved in hydrogen bonding have been omitted for clarity.

## supplementary crystallographic information

### Comment

1,2,4-Triazoles are known to possess a broad range of biological activities, finding applications as antifungal (Al-Masoudi *et al.*, 2006; Torres *et al.*, 2005), anticancer (Lin *et al.*, 2005; Demirbas *et al.*, 2002) and antiviral (Witkowski *et al.*, 1972; Aoyama *et al.*,1984) activities. Besides, these derivatives have shown herbicidal, anticonvulsant and hypocholesteramic activities.

As part of our on going research on the synthesis of biologically active heterocyclic compounds (Akhtar *et al.*, 2006, 2007, 2008), the title compound (I) was synthesized by cyclodehydration of 4-*n*-butyl-1-(3-methylbenzoyl) semicarbazide under basic conditions (Akhtar *et al.*, 2006) to evaluate its biological activities.

The two rings enclose a dihedral angle of 56.63 (13)°. There is an intermolecular N—H···O=C hydrogen bond between the amino hydrogen and the carbonyl oxygen giving rise to a zigzag chain of molecules parallel to the *c* axis (Fig. 2).

### Experimental

A solution of 4-*n*-butyl-1-(3-methylbenzoyl) semicarbazide (0.30 g) and aqueous sodium hydroxide (5%, 30 ml) was refluxed for a period of five hours till the complete consumption of semicarbazide. The reaction mixture was cooled to room temperature and neutralized with 6*M* HCl. The precipitated solid was filtered, washed with excess water, dried and recrystallized from aqueous ethanol to afford pure crystalline 4-*n*-butyl-5-(3-methyl phenyl)-2*H*-1,2,4-triazol-3(4*H*)-one.

### Refinement

In the absence of anomalous scatterers Friedel pairs were merged. H atoms bonded were included in the refinements at geometrically idealized positions with aromatic and methyl C—H distances 0.95 and 0.96Å, respectively, and U_iso_(H) values of 1.2U_eq_ of the atoms to which they were bonded. The methyl groups were allowed to rotate but not to tip. The H atom bonded to N was freely refined. The final difference map was free of any chemically significant features.

### Crystal data

**Table:**

C_13_H_17_N_3_O	*F*_000_ = 992
*M**_r_* = 231.30	*D*_x_ = 1.230 Mg m^−^^3^
Orthorhombic, *C**c**c*2	Mo *K*α radiation λ = 0.71073 Å
Hall symbol: C 2 -2c	Cell parameters from 1751 reflections
*a* = 16.905 (5) Å	θ = 3.0–22.2º
*b* = 18.139 (5) Å	µ = 0.08 mm^−^^1^
*c* = 8.145 (2) Å	*T* = 296 (2) K
*V* = 2497.5 (11) Å^3^	Orthorhombic, white
*Z* = 8	0.26 × 0.19 × 0.12 mm

### Data collection

**Table:**

Bruker APEXII CCD area-detector diffractometer	1791 independent reflections
Radiation source: fine-focus sealed tube	1118 reflections with *I* > 2σ(*I*)
Monochromator: graphite	*R*_int_ = 0.066
Detector resolution: 7.40 pixels mm^-1^	θ_max_ = 29.2º
*T* = 296(2) K	θ_min_ = 2.4º
φ and ω scans	*h* = −20→23
Absorption correction: none	*k* = −23→24
8129 measured reflections	*l* = −11→7

### Refinement

**Table:**

Refinement on *F*^2^	Secondary atom site location: difference Fourier map
Least-squares matrix: full	Hydrogen site location: inferred from neighbouring sites
*R*[*F*^2^ > 2σ(*F*^2^)] = 0.046	H atoms treated by a mixture of independent and constrained refinement
*wR*(*F*^2^) = 0.106	*w* = 1/[σ^2^(*F*_o_^2^) + (0.0435*P*)^2^] where *P* = (*F*_o_^2^ + 2*F*_c_^2^)/3
*S* = 1.07	(Δ/σ)_max_ < 0.001
1791 reflections	Δρ_max_ = 0.15 e Å^−^^3^
160 parameters	Δρ_min_ = −0.25 e Å^−^^3^
1 restraint	Extinction correction: none
Primary atom site location: structure-invariant direct methods	

### Special details

**Table:**

Geometry. All e.s.d.'s (except the e.s.d. in the dihedral angle between two l.s. planes) are estimated using the full covariance matrix. The cell e.s.d.'s are taken into account individually in the estimation of e.s.d.'s in distances, angles and torsion angles; correlations between e.s.d.'s in cell parameters are only used when they are defined by crystal symmetry. An approximate (isotropic) treatment of cell e.s.d.'s is used for estimating e.s.d.'s involving l.s. planes.
Refinement. Refinement of *F*^2^ against ALL reflections. The weighted *R*-factor *wR* and goodness of fit *S* are based on *F*^2^, conventional *R*-factors *R* are based on *F*, with *F* set to zero for negative *F*^2^. The threshold expression of *F*^2^ > σ(*F*^2^) is used only for calculating *R*-factors(gt) *etc*. and is not relevant to the choice of reflections for refinement. *R*-factors based on *F*^2^ are statistically about twice as large as those based on *F*, and *R*- factors based on ALL data will be even larger.

### Fractional atomic coordinates and isotropic or equivalent isotropic displacement parameters (Å^2^)

**Table:**

	*x*	*y*	*z*	*U*_iso_*/*U*_eq_	
N1	0.09066 (14)	0.37056 (10)	0.2364 (3)	0.0349 (5)	
N2	0.08503 (15)	0.41720 (12)	−0.0051 (3)	0.0407 (6)	
N3	0.08408 (14)	0.34149 (11)	−0.0273 (3)	0.0412 (6)	
O1	0.09681 (11)	0.49894 (11)	0.2110 (2)	0.0500 (6)	
C1	0.08634 (17)	0.23582 (14)	0.1565 (3)	0.0350 (6)	
C2	0.02844 (17)	0.20574 (14)	0.2574 (4)	0.0449 (8)	
H2	−0.0090	0.2359	0.3072	0.054*	
C3	0.0272 (2)	0.13041 (15)	0.2829 (4)	0.0531 (9)	
H3	−0.0111	0.1100	0.3510	0.064*	
C4	0.08179 (19)	0.08554 (16)	0.2092 (4)	0.0501 (8)	
H4	0.0791	0.0349	0.2257	0.060*	
C5	0.14059 (18)	0.11403 (14)	0.1111 (4)	0.0423 (7)	
C6	0.14204 (17)	0.18975 (14)	0.0851 (3)	0.0389 (7)	
H6	0.1811	0.2099	0.0185	0.047*	
C7	0.08757 (16)	0.31523 (14)	0.1205 (3)	0.0355 (6)	
C8	0.09113 (17)	0.43677 (14)	0.1525 (3)	0.0360 (6)	
C9	0.11284 (18)	0.36397 (14)	0.4094 (3)	0.0385 (7)	
H9A	0.0902	0.3192	0.4546	0.046*	
H9B	0.0916	0.4055	0.4701	0.046*	
C10	0.20182 (18)	0.36205 (16)	0.4290 (4)	0.0475 (7)	
H10A	0.2223	0.3202	0.3683	0.057*	
H10B	0.2239	0.4063	0.3803	0.057*	
C11	0.22993 (18)	0.35668 (17)	0.6045 (4)	0.0499 (8)	
H11A	0.2064	0.3137	0.6557	0.060*	
H11B	0.2125	0.3999	0.6646	0.060*	
C12	0.2024 (2)	0.06501 (17)	0.0313 (5)	0.0615 (10)	
H13A	0.2245	0.0325	0.1123	0.092*	
H13B	0.2436	0.0951	−0.0145	0.092*	
H13C	0.1782	0.0364	−0.0543	0.092*	
H22	0.0837 (17)	0.4487 (16)	−0.097 (4)	0.060 (10)*	
C13	0.31893 (19)	0.35095 (19)	0.6144 (5)	0.0622 (9)	
H13D	0.3366	0.3098	0.5500	0.093*	
H13E	0.3345	0.3440	0.7267	0.093*	
H13F	0.3423	0.3955	0.5730	0.093*	

### Atomic displacement parameters (Å^2^)

**Table:**

	*U*^11^	*U*^22^	*U*^33^	*U*^12^	*U*^13^	*U*^23^
N1	0.0461 (15)	0.0309 (11)	0.0277 (12)	−0.0011 (10)	−0.0020 (12)	−0.0027 (9)
N2	0.0577 (17)	0.0319 (12)	0.0325 (14)	−0.0033 (11)	−0.0061 (12)	0.0009 (10)
N3	0.0553 (15)	0.0328 (12)	0.0356 (14)	−0.0028 (10)	−0.0050 (12)	−0.0019 (11)
O1	0.0770 (15)	0.0307 (9)	0.0422 (12)	−0.0055 (11)	−0.0009 (11)	−0.0052 (9)
C1	0.0398 (16)	0.0312 (13)	0.0341 (15)	−0.0004 (12)	−0.0074 (13)	−0.0035 (12)
C2	0.047 (2)	0.0412 (15)	0.0464 (18)	−0.0008 (12)	0.0070 (15)	−0.0014 (14)
C3	0.056 (2)	0.0449 (16)	0.058 (2)	−0.0091 (15)	0.0099 (16)	0.0072 (15)
C4	0.063 (2)	0.0333 (14)	0.054 (2)	−0.0028 (14)	−0.0098 (18)	0.0041 (15)
C5	0.0478 (18)	0.0347 (14)	0.0444 (17)	0.0043 (13)	−0.0105 (16)	−0.0025 (14)
C6	0.0432 (17)	0.0385 (15)	0.0350 (16)	−0.0024 (12)	−0.0011 (13)	−0.0017 (13)
C7	0.0373 (16)	0.0332 (13)	0.0361 (16)	−0.0021 (11)	−0.0073 (14)	−0.0013 (11)
C8	0.0434 (17)	0.0343 (14)	0.0303 (15)	−0.0015 (12)	−0.0013 (13)	−0.0029 (12)
C9	0.0486 (19)	0.0365 (14)	0.0303 (15)	−0.0052 (12)	0.0002 (13)	−0.0013 (12)
C10	0.052 (2)	0.0548 (17)	0.0358 (16)	−0.0006 (14)	−0.0003 (15)	−0.0009 (13)
C11	0.057 (2)	0.0537 (18)	0.0393 (17)	0.0043 (15)	−0.0044 (16)	0.0027 (14)
C12	0.068 (3)	0.0503 (18)	0.066 (2)	0.0181 (17)	−0.0004 (19)	−0.0076 (17)
C13	0.059 (2)	0.069 (2)	0.059 (2)	0.0085 (17)	−0.012 (2)	−0.0021 (18)

### Geometric parameters (Å, °)

**Table:**

N1—C7	1.379 (3)	C5—C12	1.518 (4)
N1—C8	1.382 (3)	C6—H6	0.9300
N1—C9	1.463 (3)	C9—C10	1.513 (4)
N2—C8	1.336 (4)	C9—H9A	0.9700
N2—N3	1.385 (3)	C9—H9B	0.9700
N2—H22	0.94 (3)	C10—C11	1.509 (4)
N3—C7	1.296 (3)	C10—H10A	0.9700
O1—C8	1.228 (3)	C10—H10B	0.9700
C1—C6	1.387 (4)	C11—C13	1.510 (4)
C1—C2	1.390 (4)	C11—H11A	0.9700
C1—C7	1.470 (4)	C11—H11B	0.9700
C2—C3	1.382 (3)	C12—H13A	0.9600
C2—H2	0.9300	C12—H13B	0.9600
C3—C4	1.369 (4)	C12—H13C	0.9600
C3—H3	0.9300	C13—H13D	0.9600
C4—C5	1.376 (4)	C13—H13E	0.9600
C4—H4	0.9300	C13—H13F	0.9600
C5—C6	1.390 (4)		
			
C7—N1—C8	107.1 (2)	N1—C9—C10	111.0 (2)
C7—N1—C9	127.6 (2)	N1—C9—H9A	109.4
C8—N1—C9	123.1 (2)	C10—C9—H9A	109.4
C8—N2—N3	113.0 (2)	N1—C9—H9B	109.4
C8—N2—H22	127.2 (19)	C10—C9—H9B	109.4
N3—N2—H22	119.8 (19)	H9A—C9—H9B	108.0
C7—N3—N2	104.0 (2)	C11—C10—C9	114.5 (3)
C6—C1—C2	119.3 (2)	C11—C10—H10A	108.6
C6—C1—C7	119.8 (3)	C9—C10—H10A	108.6
C2—C1—C7	120.8 (3)	C11—C10—H10B	108.6
C3—C2—C1	119.2 (3)	C9—C10—H10B	108.6
C3—C2—H2	120.4	H10A—C10—H10B	107.6
C1—C2—H2	120.4	C10—C11—C13	111.7 (3)
C4—C3—C2	120.8 (3)	C10—C11—H11A	109.3
C4—C3—H3	119.6	C13—C11—H11A	109.3
C2—C3—H3	119.6	C10—C11—H11B	109.3
C3—C4—C5	121.2 (3)	C13—C11—H11B	109.3
C3—C4—H4	119.4	H11A—C11—H11B	108.0
C5—C4—H4	119.4	C5—C12—H13A	109.5
C4—C5—C6	118.2 (3)	C5—C12—H13B	109.5
C4—C5—C12	121.7 (3)	H13A—C12—H13B	109.5
C6—C5—C12	120.1 (3)	C5—C12—H13C	109.5
C5—C6—C1	121.3 (3)	H13A—C12—H13C	109.5
C5—C6—H6	119.3	H13B—C12—H13C	109.5
C1—C6—H6	119.3	C11—C13—H13D	109.5
N3—C7—N1	111.7 (2)	C11—C13—H13E	109.5
N3—C7—C1	123.0 (2)	H13D—C13—H13E	109.5
N1—C7—C1	125.2 (2)	C11—C13—H13F	109.5
O1—C8—N2	128.5 (3)	H13D—C13—H13F	109.5
O1—C8—N1	127.4 (2)	H13E—C13—H13F	109.5
N2—C8—N1	104.1 (2)		
			
C8—N2—N3—C7	1.7 (3)	C9—N1—C7—C1	16.6 (4)
C6—C1—C2—C3	−0.6 (4)	C6—C1—C7—N3	55.9 (4)
C7—C1—C2—C3	177.3 (3)	C2—C1—C7—N3	−122.1 (3)
C1—C2—C3—C4	−0.5 (5)	C6—C1—C7—N1	−125.7 (3)
C2—C3—C4—C5	1.7 (5)	C2—C1—C7—N1	56.4 (4)
C3—C4—C5—C6	−1.6 (5)	N3—N2—C8—O1	176.6 (3)
C3—C4—C5—C12	178.8 (3)	N3—N2—C8—N1	−2.6 (3)
C4—C5—C6—C1	0.5 (4)	C7—N1—C8—O1	−176.8 (3)
C12—C5—C6—C1	−179.9 (3)	C9—N1—C8—O1	−12.7 (5)
C2—C1—C6—C5	0.7 (4)	C7—N1—C8—N2	2.5 (3)
C7—C1—C6—C5	−177.3 (3)	C9—N1—C8—N2	166.6 (3)
N2—N3—C7—N1	0.0 (3)	C7—N1—C9—C10	80.2 (3)
N2—N3—C7—C1	178.7 (3)	C8—N1—C9—C10	−80.5 (3)
C8—N1—C7—N3	−1.6 (3)	N1—C9—C10—C11	178.9 (2)
C9—N1—C7—N3	−164.8 (3)	C9—C10—C11—C13	177.0 (2)
C8—N1—C7—C1	179.8 (3)		

### Hydrogen-bond geometry (Å, °)

**Table:**

*D*—H···*A*	*D*—H	H···*A*	*D*···*A*	*D*—H···*A*
N2—H22···O1^i^	0.94 (3)	1.84 (3)	2.775 (3)	170 (3)

Symmetry codes: (i) *x*, −*y*+1, *z*−1/2.
